# Whole transcriptome analysis of the differential RNA profiles and associated competing endogenous RNA networks in LPS-induced acute lung injury (ALI)

**DOI:** 10.1371/journal.pone.0251359

**Published:** 2021-05-07

**Authors:** Xiangnan Teng, Jing Liao, Lili Zhao, Wei Dong, Haiyi Xue, Lang Bai, Shanling Xu

**Affiliations:** 1 School of Medicine, University of Electronic Science and Technology of China, Chengdu, China; 2 Department of Critical Care Medicine, Sichuan Cancer Hospital & Institute, Sichuan Cancer Center, School of Medicine, University of Electronic Science and Technology of China, Chengdu, China; 3 Center of Infectious Diseases, West China Hospital of Sichuan University, Chengdu, China; Texas Tech Health Sciences Center El Paso, UNITED STATES

## Abstract

Acute lung injury (ALI) is a serious inflammation disease usually arises alveolar epithelial membrane dysfunction and even causes death. Therefore, the aims of this study are to screen the differentially expressed lncRNAs, circRNAs, miRNAs, and mRNAs in ALI based on the high-throughput sequencing. The lipopolysaccharide (LPS)-induced ALI mouse model was established, the injury of ALI mouse model was evaluated through histological analysis with hemotoxylin and eosin (H & E) staining assay, dry/wet ratio, infiltrated-immune cells, ET-1 mRNA expression and released-proinflammation factors. Then, expression data of lncRNAs, circRNAs, miRNAs and mRNAs in ALI were acquired using whole-transcriptome sequencing. The differential expression of lncRNAs (DE lncRNAs), circRNAs (DE circRNAs), miRNAs (DE miRNAs) and mRNAs (DE mRNAs) were identified, and the lncRNA-miRNA-mRNA network and circRNA-miRNA-mRNA network were constructed, and the biological function of target genes were annotated based on bioinformatics analysis. In the present study, the LPS-induced ALI mouse model was successfully established. The biological analysis results showed that total 201 DE lncRNAs, 172 DE circRNAs, 62 DE miRNAs, and 3081 DE mRNAs were identified in ALI. The 182 lncRNA-miRNA-mRNA networks and 32 circRNA-miRNA-mRNA networks were constructed were constructed based on the correlation between lncRNAs/circRNAs, miRNAs, mRNAs. The biological function analysis indicated that TNF signaling pathway, chemokine signaling pathway and so on involved in ALI. In the present study, the differential expression coding and non-coding RNAs (ncRNAs) in ALI were identified, and their regulatory networks were constructed. There might provide the potential biomarkers and underlying mechanism for ALI diagnosis and treatment.

## Introduction

Acute lung injury (ALI) has defined as a moderate or mild acute respiratory distress syndrome (ARDS) in the past 50 years [1]. It also relates to significant morbidity and high mortality in the ALI patients with complex and cascading development processes [2]. The histological characteristics of ALI include neutrophilic alveolitis, alveolar epithelial and endothelial damage, the formation of hyaline membrane, and microvascular thrombosis [3, 4]. The pathogenesis of ALI has been found, including acute inflammation that induces lung endothelial and epithelial barriers disruption [5]. Understanding the pathogenesis of ALI is vital for improvement and treatment LPS-induced lung injury.

Recent years, non-coding RNAs (ncRNAs) emerge the key role in cell function, for instance, rRNAs, tRNAs, small nuclear RNA (snRNAs), small nucleolar RNAs (snoRNAs) involve in the mRNA translation, splicing, modification of rRNAs, respectively [6]. Mostly, ncRNAs regulate mRNA stability through modulating the transcriptional or post-transcriptional expression via a regulatory network [7]. Based on the next generation sequencing (NGS) technologies and bioinformatics analysis, a plenty of lncRNAs, circRNAs, miRNAs, mRNAs and their network have been discovered [8]. Long-noncoding RNAs (LncRNAs) is a class of ncRNAs with more than 200 nt length that function with or without protein-coding capacity [9]. Aberrant expression of lncRNAs associate with ALI, such as, lncRNA TUG1 has been demonstrated improving sepsis-induced ALI by targeting miR-34b-5p to increase GAB1 expression [10]. In addition, lncRNA CASC2 alleviates lipopolysaccharide-induced ALI via regulating miR-27b/TAB2 axis [11]. Chen H, et al have indicated that lncRNA THRIL enhances sepsis-induced ALI through modulating miR-424/ROCK2 axis [12]. circRNAs is a group of ncRNAs that widely express in eukaryotic cells with the covalent closed circular structure without 5’ and 3’ polarities and poly A tail configuration [13]. CricRNAs always act as spongers for miRNAs to regulate the function of miRNAs [14]. The regulatory effects of circRNAs on multiple diseases such as diabetes mellitus, neurological disorders, cardiovascular, cancers and some immune diseases [15]. For example, circ_0054633 promotes inflammation and proliferation in LPS-induced ALI [16]. Mus musculus (mmu)_circ_0001679, mmu_circ_0001212 have been identified as therapeutic targets for attenuating the sepsis-induced ALI [17]. Despite several lncRNAs and circRNAs have been identified in ALI, however, there are largely lncRNAs and circRNAs remain unclear.

Taken together, in the present study, whole-transcriptome sequencing was performed to screen the differently expressed lncRNAs, circRNAs, miRNAs, and mRNAs. Then, based on the bioinformatics analysis, the lncRNA-miRNA-mRNA networks and circRNA-miRNA-mRNA networks were constructed, and the biological function of target genes were identified.

## Material and methods

### Construction of the ALI mouse model

The ALI mouse model was established according to the previous described [18]. All animal experiments in this study were approved by Ethics Committee of Sichuan Cancer Hospital (SCCHEC-04-2020-002), and the animal experiments were archived following the animal care guidelines. A total six male C57BL/6 mice (six to eight-week-old, 28–22 g) were purchased from the Experimental Animal Center of Kunming Medical University. Mice were randomly divided into two groups, control group and LPS-induced ALI group. After anesthetizing with intraperitoneal injection of pentobarbital sodium (20 g/140 μL), mice were exposed the trachea and intra-tracheally accepted the LPS administration (25mg/kg) to establish the LPS-induced ALI group mice. In addition, the control group mice were injected with equal volume of intra-tracheal saline administration. After 12 h, mice were euthanatized then blood and lung tissues were harvested for subsequent experiments. After that, the ALI mouse model was evaluated that based on histological change, lung permeability change, inflammation activation.

### Histological examination

The right lung tissues were fixed with 4% paraformaldehyde (PFA) and embedded in paraffin. Then, the sections were sliced into 4-μm slices and dewaxed with xylene, dehydrated with increasing concentration of ethanol. The slices were stained with H & E solution. The lung injury was observed and photographed under an optical microscope. Lung injury score was assessed according to the previous described [19].

### Neutrophils counting

After mice were euthanatized, then weighed the left lung tissues, left tissues were used for bronchoalveolar lavage (BAL). After bronchoalveolar lavage fluid (BALF) was collected and centrifuged, the precipitation was diluted with 1 mL saline to count neutrophils.

### Lung wet/dry (W/D) ratio

The lung edema was assessed by W/D ration. After 12 h of LPS stimulated, mice were euthanatized and left lung tissue was harvested and immediately weighed, and then was dried in an incubated at 60°C for 48 h and subsequently weighed.

### RNA extraction and quantitative PCR

Total RNA was extracted from tissues using Trizol reagent (Takara, Dalian, China) according to the manufacturer’s suggestion. RNA was transcribed into cDNAs by the Prime Script^™^ II Reverse Transcriptase (Takara) following the suggestion of manufacturer. QPCR was performed using SYBR^®^ Premix Ex Taq^™^ II (Takara) obeyed the protocol of manufacturer in a Step One Plus^™^ Real‐Time PCR System (ABI, Applied Biosystems, Foster City, CA). The expression of genes was calculated by 2^-ΔΔCt^ methods. All experiments were performed independently for three times. GAPDH was used to normalize the expression of genes in this study. All primers were listed as following, ET-1, F, 5’-GGGGTTCGAACTTGGAGCAG-3’, R, 5’- CAGAGGACATGCCGAATCCA-3’. GAPDH, F, 5’-GCTCCCTCTTTCTTTGCAGC-3’, R, 5’-ACCATGAGTCCTTCCACGAT-3’.

### Enzyme-linked immunosorbent assay (ELISA) measurement

Inflammation factors, including Concentrations of Myeloperoxidase (MPO), Tumor Necrosis Factor (TNF-α), Interleukin-1β (IL-1β), and Interleukin-6 (IL-6) of lung tissues were analyzed using ELISA kits (SPECTCA, Molecular, USA).

### Sample processing and whole transcriptome sequencing

Total RNA of each mouse was extracted from lung tissues, the concentrations and purity were calculated using a NanoPhotometer (Invitrogen). The processing included RNA quality control, RNA-seq library preparation, PCR amplification and primary quantitative. Specially, the cDNA library of miRNA was established using the small RNA sample pre kit (Illumina, San Diego, CA, USA) following the manufacturer’s protocol. And the libraries were primarily quantified with Qubit2.0 and accurately quantified with qPCR. cDNA library of circRNA was established after ribosomal RNA (rRNA) removal using the Ribo-Zero^™^ Magnetic Kit (Epicentre Technologies, Madison, WI, USA) and linear RNA removal with RNase R (Epicentre Technologies) treatment following the manufacturer’s protocol. Then, the libraries were constructed using NEBNext Ultra RNA Library Prep Kit for Illumina (NEB, USA) according to the protocol of manufacturer. RNA quality control including detection of DNA contamination, RNA purity, and integration of RNA size were examined using agarose gel electrophoresis, NanoPhotometer, Agilent 2100 bioanalyzer, respectively. Non-DNA contamination, OD260/OD280 ratio between 1.8–2.0 and 260/230 ratio between 1.8–2.2, 28 S/18 S ratio > 1.5, and RNA Integrity Number (RIN) > 7 were used as the criterion of high-quality RNA. After RNA quality control and RNA-seq library preparation, the libraries were quantified using KAPA Library Quantification kit (KAPA Biosystems, South Africa). Finally, the paired-end sequencing of individual libraries was performed on an Illumina HiSeq sequencer (Illumina).

### Bioinformation analysis

The raw reads presented some adaptor sequences or low-quality of reads. Therefore, the raw reads were cleaned and filtered to obtain the high-quality reads. The clean reads were mapped to reference sequences using HISAT2, and spliced using Stringtie. The differential expression genes (DEGs), lncRNAs (DE lncRNAs), circRNAs (DE circRNAs), miRNAs (DE miRNAs) were analyzed Limma R software [20]. Biological function for genes were performed using KOBAS (2.0) [21].

### DEGs, DE lncRNAs, DE circRNAs, DE miRNAs analysis

The DEGs and DE lncRNAs between ALI mice and control mice were analyzed using ballgown package with the cutoff criterion with adj. *P*<0.05 and |log2 fold change (FC)|>2. The DE miRNAs and DE circRNAs between ALI mice and control mice were determined using DESeq2 package [22] with threshold of adj. *P*<0.05 and |log2 (FC)|>2.

### Hierarchical clustering analysis

The clustering of DEGs, DE lncRNAs, D EmiRNAs and DEcircRNAs was performed using hierarchical clustering, K-means, and SOM clustering package.

### Gene ontology (GO) and Kyoto Encyclopedia of Genes and Genomes (KEGG) enrichment analysis

GO enrichment (cellular component, CC; biological process, BP; molecular function, MF) was performed using GOseq package with threshold with adj. *P*<0.05. And the KEGG pathway enrichment was determined using KOBAS (2.0) software with cutoff value of adj. *P*<0.05 and FDR≤0.05.

### Correlation analysis of circRNAs, lncRNAs, miRNAs and mRNA in ALI

The target genes of lncRNAs were predicated using Pearson correlation analysis with | coefficient value |>0.95. Moreover, the speculated target genes of miRNAs were predicated using miRanda, PITA and RNAhybrid software. In addition, the binding sites of circRNAs at miRNAs were predicated using miRanda software.

### Construction of lncRNA-miRNA-mRNA, and circRNA-miRNA-mRNA co-expression network

The lncRNA-miRNA-mRNA (ceRNA) network and circRNA-miRNA-mRNA (ceRNA) network were constructed according to the lncRNA-miRNA-gene pairs and circRNA-miRNA-gene pairs, and visualized Cytoscape v3.6.0.

### Statistical analysis

Data in this study presented as mean ± standard deviation (SD). Comparison differences between two groups or multiple groups were examined using Student’s *t*-test or one-way analysis of variance (ANOVA). *P* value < 0.05 was considered as statistical significance.

## Results

### Established of LPS-induced ALI mouse model

**Fig 1:**
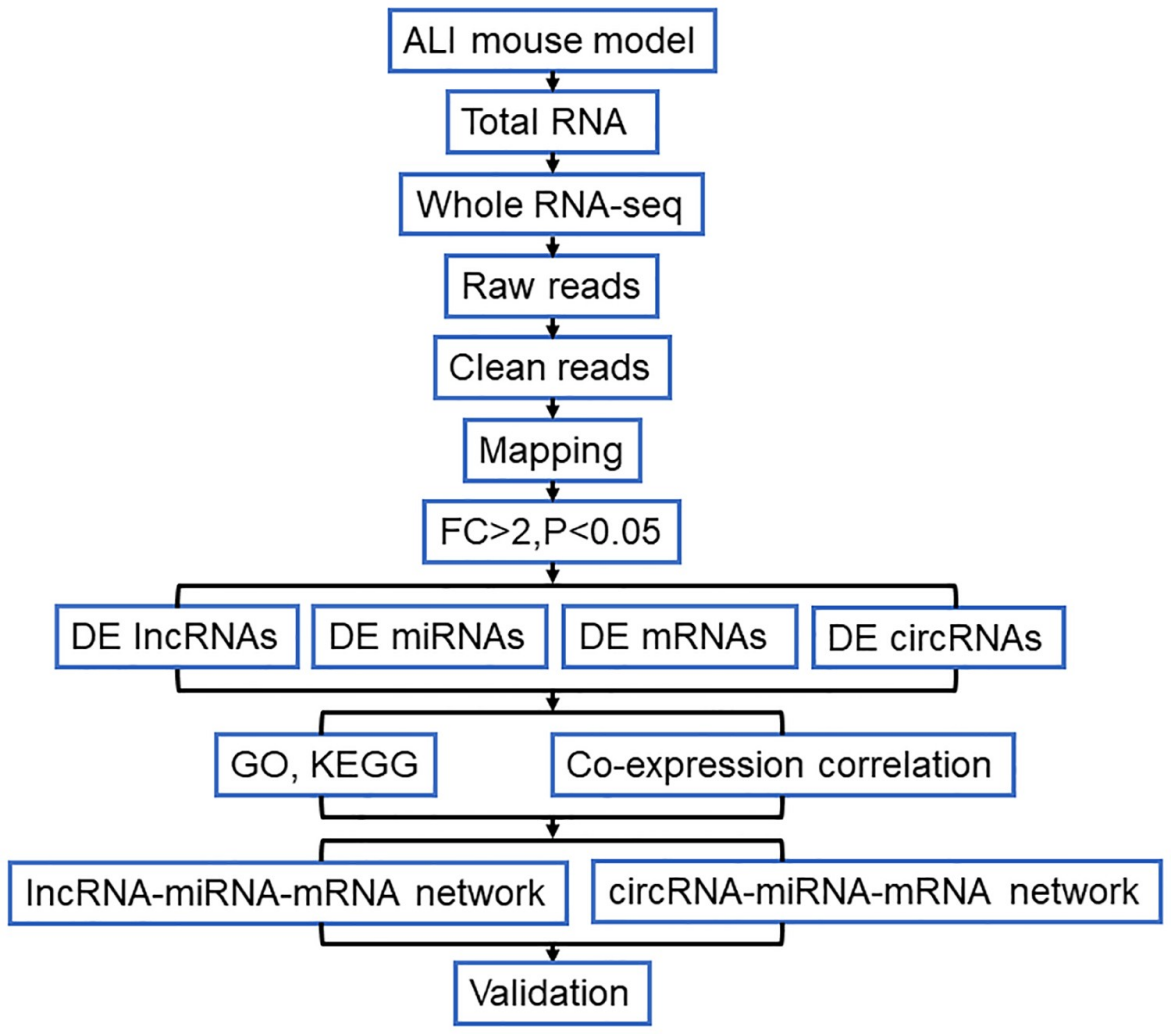
Flowchart for analysis of the whole-transcriptome sequencing analysis.

**Fig 2:**
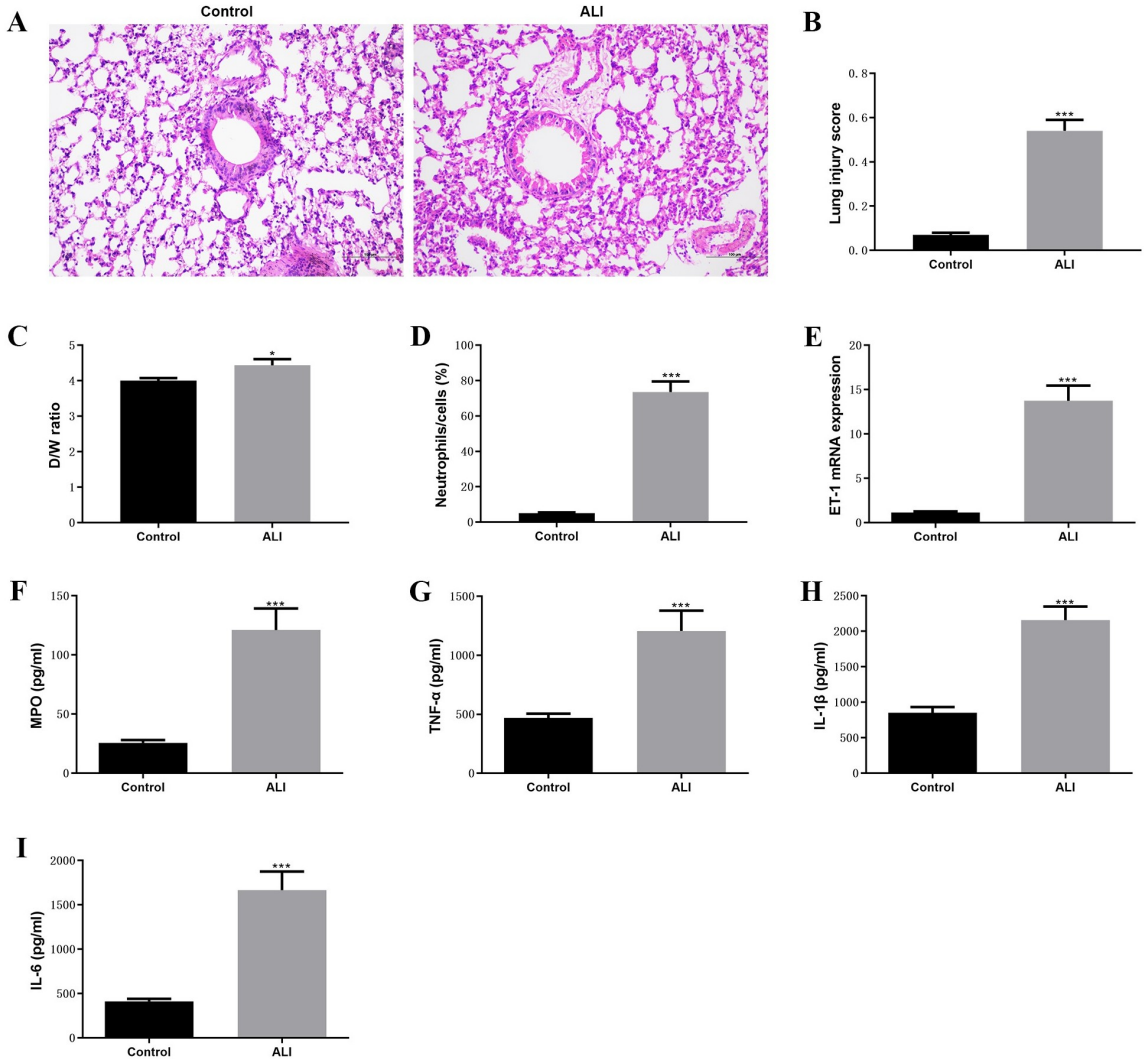
Established of LPS-induced ALI mouse model. (A)-(D) The histology of lung tissue calculated using H & E staining, lung injury score, D/W ratio, and infiltrated neutrophils between ALI and control groups, respectively. (E) The mRNA expression of ET-1 between ALI and control groups were determined by RT-qPCR. (F)-(I) The levels of MPO, TNF-α, IL-1β, and IL-6 between ALI and control groups were detected by ELISA assay. ***P<0.001.

The purpose of this study is to explore the novel molecular biomarkers for ALI. Therefore, the LPS-induced ALI mouse model was established and the whole transcriptome was analyzed in this study. The analysis strategy and procedure of the present study has shown in Fig 1. Firstly, the LPS-induced ALI mouse model was constructed and evaluated according to the previous description [19]. The histological results indicated that the increasing inflammatory cells infiltrated into the alveolar, type I and II alveolar epithelial cells significantly swelled, and the alveolar basement membranes were ruptured comparted to control group (Fig 2A). Obviously, the high lung injury score was observed compared with the control group (Fig 2B). In addition, the inflammation of lung tissues was examined, we found that W/D ratio of ALI mouse higher than the W/D ration of control mouse (Fig 2C). Besides, the number of neutrophils remarkably increased in ALI group compared with control group (Fig 2D). The RT-qPCR results indicated that the expression of ET-1 significantly upregulated in ALI compared to control group (Fig 2E). Then, the pro-inflammatory factors were detected by ELISA, increasing concentration of MOP, TNF-α, IL-1β, and IL-6 was observed in ALI group compared with control group (Fig 2F–2I). These results suggested that the successful establishment of the LPS-induced ALI mouse model.

### Identification of DE lncRNAs, DE mRNAs, DE circRNAs, DE miRNAs in ALI mouse model

Next, the whole transcriptome sequencing was performed by the Illumina Hiseq2500 platform, the clean reads of ALI group samples were mapped and the clean readers were classified into different ratio of protein coding, miRNA, lincRNA, antisense, processed transcript, processed pseudogene, and transcribed unprocessed pseudogene and others in ALI group. And the clean readers generated from control group samples were classified into different ratio of protein coding, miRNA, lincRNA, antisense, processed pseudogene, processed transcript, mt rRNA, IG C/V gene (S1 Fig).

**Fig 3:**
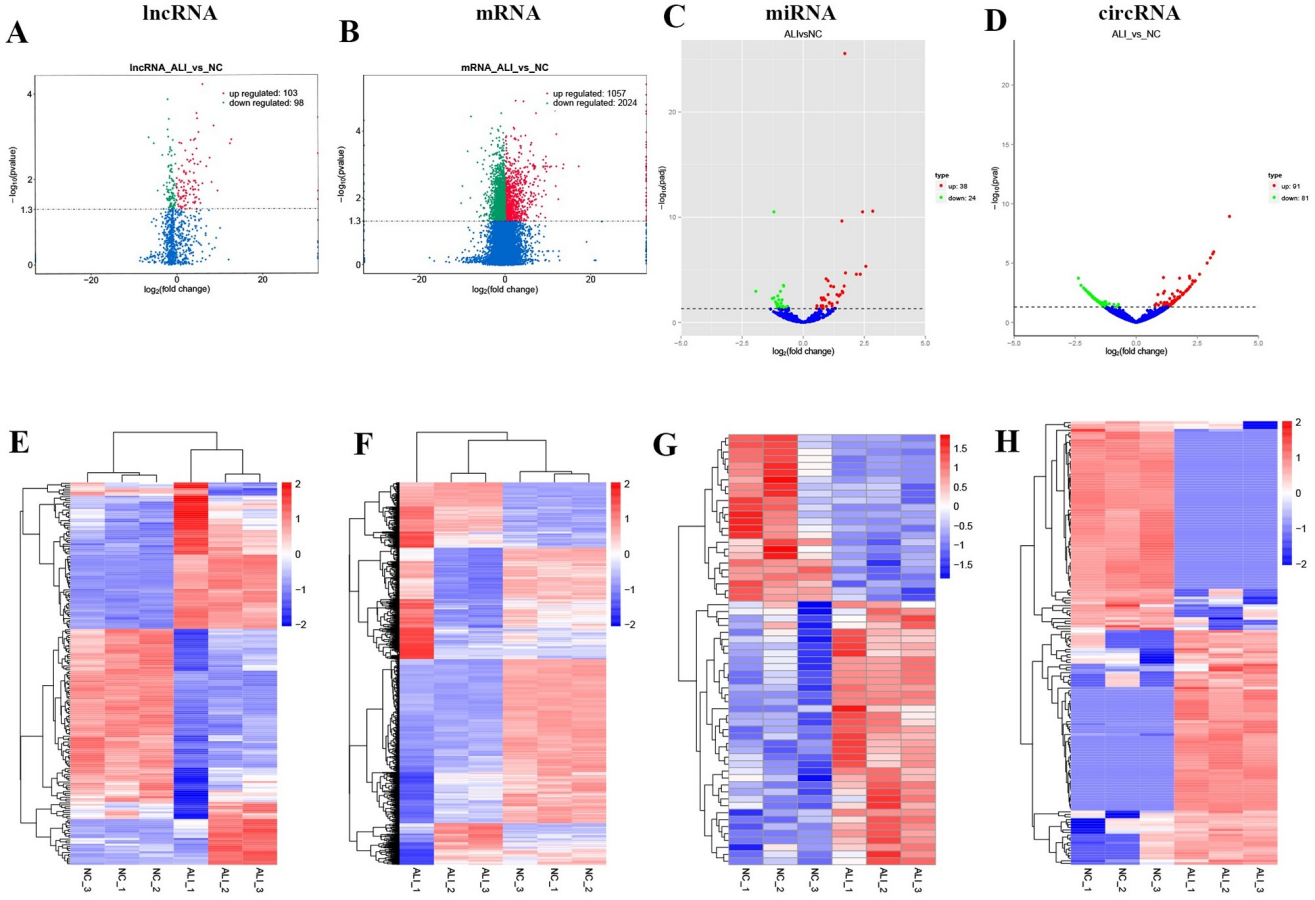
Identification of DE lncRNAs, DE mRNAs, DE circRNAs, DE miRNAs in ALI mouse model. (A)-(D) The volcano plots of DE lncRNAs, DE mRNAs, DE circRNAs, DE miRNAs in ALI mouse model. (E)-(H) The heatmap of DE lncRNAs, DE mRNAs, DE circRNAs, DE miRNAs in ALI mouse model.

The whole transcriptome sequencing data including lncRNA, circRNA, miRNA, and mRNA were obtained, and the differential expression of lncRNA, circRNA, miRNA, and mRNA were screened with threshold of |log2(FC)|>2 and *P* value < 0.05. As shown in Fig 3A and 3E, a total 201 DE lncRNAs (103 upregulated and 98 downregulated lncRNAs) were identified (S1 Table). The characteristics of lncRNA analysis shown in S2 Fig, the mean of length, and the mean number of exons, and the ORF length of annotated/novel lncRNA were shorter than mRNA. Cumulative distribution curve revealed the sequence conservation of annotated/novel lncRNA obvious lower than mRNA. Moreover, we found the intronic lncRNAs (72.8%) is the main subtype in current study than lincRNA (21.6%) and antisense lncRNA (5.6%).

In addition, a total 3081 DE mRNAs (1057 upregulated and 2024 downregulated mRNAs, Fig 3B and 3F, S2 Table), 62 DE miRNAs (38 upregulated and 24 downregulated miRNAs, Fig 3C and 3G, S3 Table), and 172 DE circRNAs (91 upregulated and 81 downregulated circRNAs, Fig 3D and 3H, S4 Table) were identified in the ALI group compared to control group. Our finding suggested that the distinctive expression of transcriptome between ALI and control group.

### Biological function analysis

**Fig 4:**
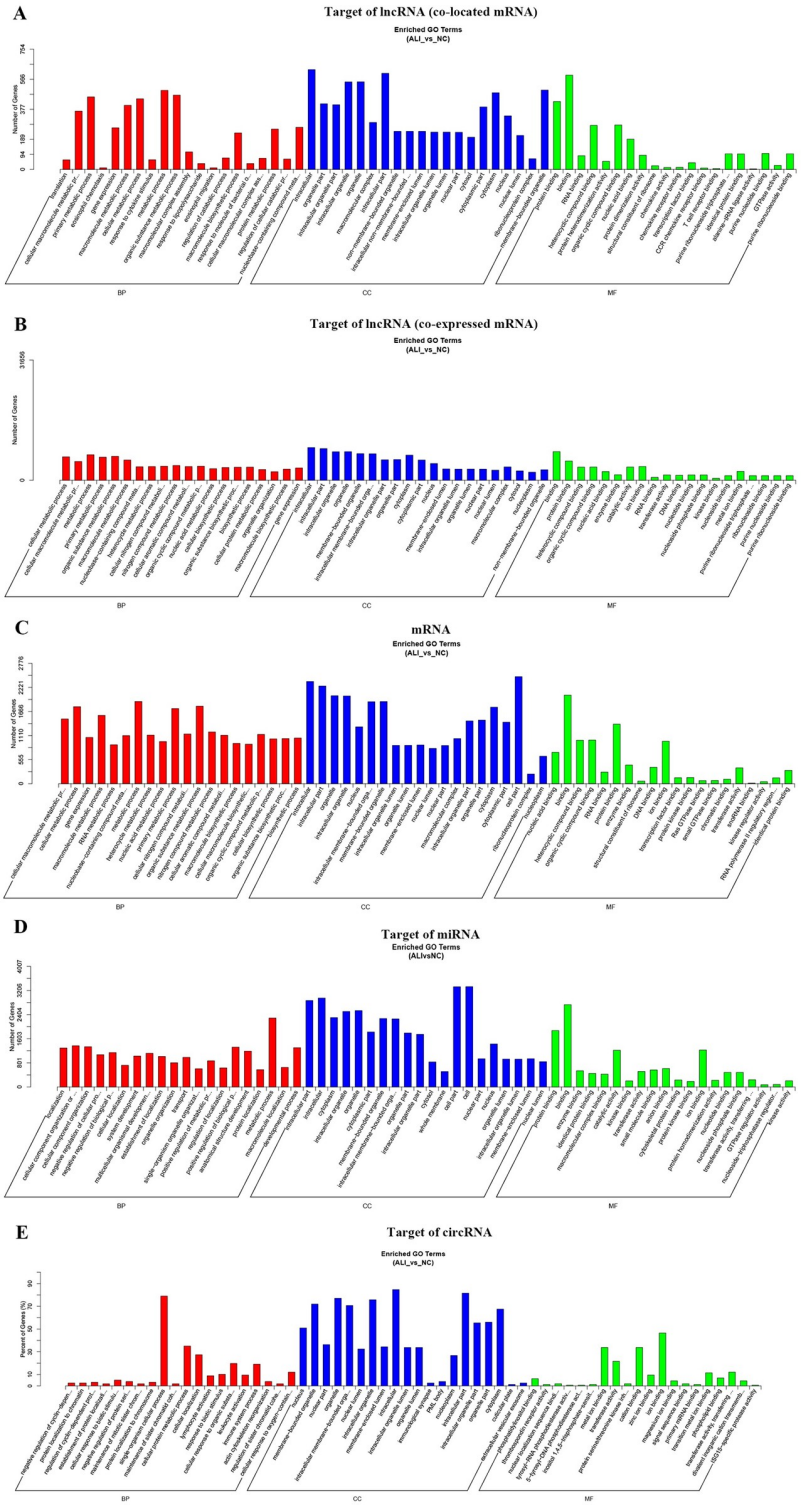
GO annotation. (A)-(D) The bar charts of GO annotation analysis for the target of lncRNAs, DE mRNAs, target of miRNAs, and target of circRNAs.

**Fig 5:**
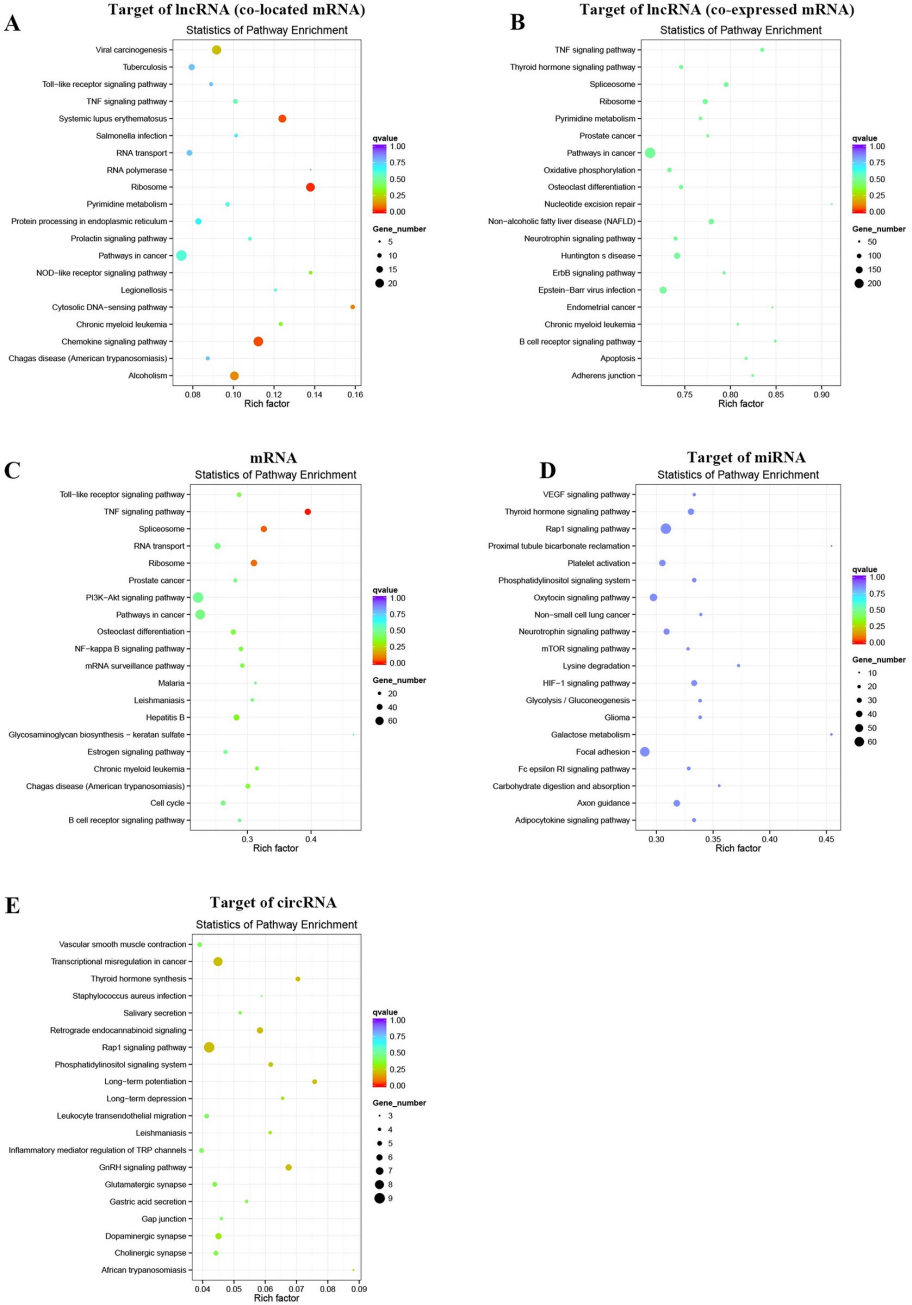
KEGG pathways enrichment. (A)-(E) The bubble plots of KEGG pathway enrichment for the target of lncRNAs, DE mRNAs, target of miRNAs, and target of circRNAs.

We further investigated the biological function of the ALI-related genes in this study. GO annotation was performed by wallenius non-central hyper-geometric distribution-based GOseq. The target genes of lncRNAs were screened according to the 100 kp upstream and downstream of lncRNAs or the cutoff of Pearson correlation coefficient ≥ 0.95 (S5 and S6 Tables). We found that target genes of DE lncRNAs were enriched in biological processes mainly enriched in metabolic processes (Fig 4A and 4B, S3 Fig, S7 and S8 Tables). The cellular component included intracellular, organelle part, macromolecular complex and so on (Fig 4A and 4B, S3 Fig, S7 and S8 Tables). And the molecular function included protein binding, RNA binding and so on (Fig 4A and 4B, S3 Fig, S7 and S8 Tables). Furthermore, KEGG pathway mainly enriched in inflammation related pathways, such as TNF signaling pathway, chemokine signaling pathway, ribosome, pathway in cancer (Fig 5A and 5B, S9 Table). In addition, mRNAs were enriched in GO-BP terms mainly included metabolic processes, GO-CC terms enriched in intracellular, organelle part, macromolecular complex and so on, and GO-MF terms included protein binding, RNA binding and so on (Fig 4C, S4A–S4C Fig, S10 Table). Moreover, the differentially expressed mRNAs enriched in TNF signaling pathway, spliceosome, ribosome and so on (Fig 5C, S11 Table).

The target genes of miRNAs were predicated by miRanda, PITA, RNAhybrid database, and overlapped by Venn diagram. Then, GO analysis indicated that target genes of miRNAs were enriched GO-BP terms including metabolic processes and so on, and GO-CC terms including intracellular, organelle part, cell part and so on, GO-MF terms including protein binding, binding and so on (Fig 4D, S4D–S4F Fig, S12 Table). The KEGG pathway enrichment analysis showed that the target genes of miRNAs were enriched in Rap1 signaling pathway, galactose metabolism, HIF-1 signaling pathway with p value < 0.05 (Fig 5D, S13 Table).

Then, the homology genes of differentially expressed circRNAs were identified and performed by GO and KEGG pathway enrichment analysis. The GO-terms enriched in single-organism cellular process, cellular protein metabolic process, and cellular localization and so on, and the GO-CC terms enriched in intracellular, intracellular part, organelle and so on, and the GO-MF terms enriched in ion binding, cation binding, metalion binding and so on (Fig 4E, S5 Fig, S14 Table). The results of KEGG pathway enrichment analysis indicated that the homology genes of differentially expressed circRNAs were enriched in GnRH signaling pathway, Transcriptional misregulation in cancer, Rap1 signaling pathway and so on (Fig 5E, S15 Table). Above results the lncRNAs, miRNAs, circRNAs-related genes were significantly enriched in metabolism processes and binding function.

### Construction of lncRNA-miRNA-mRNA network in ALI mouse model

**Fig 6:**
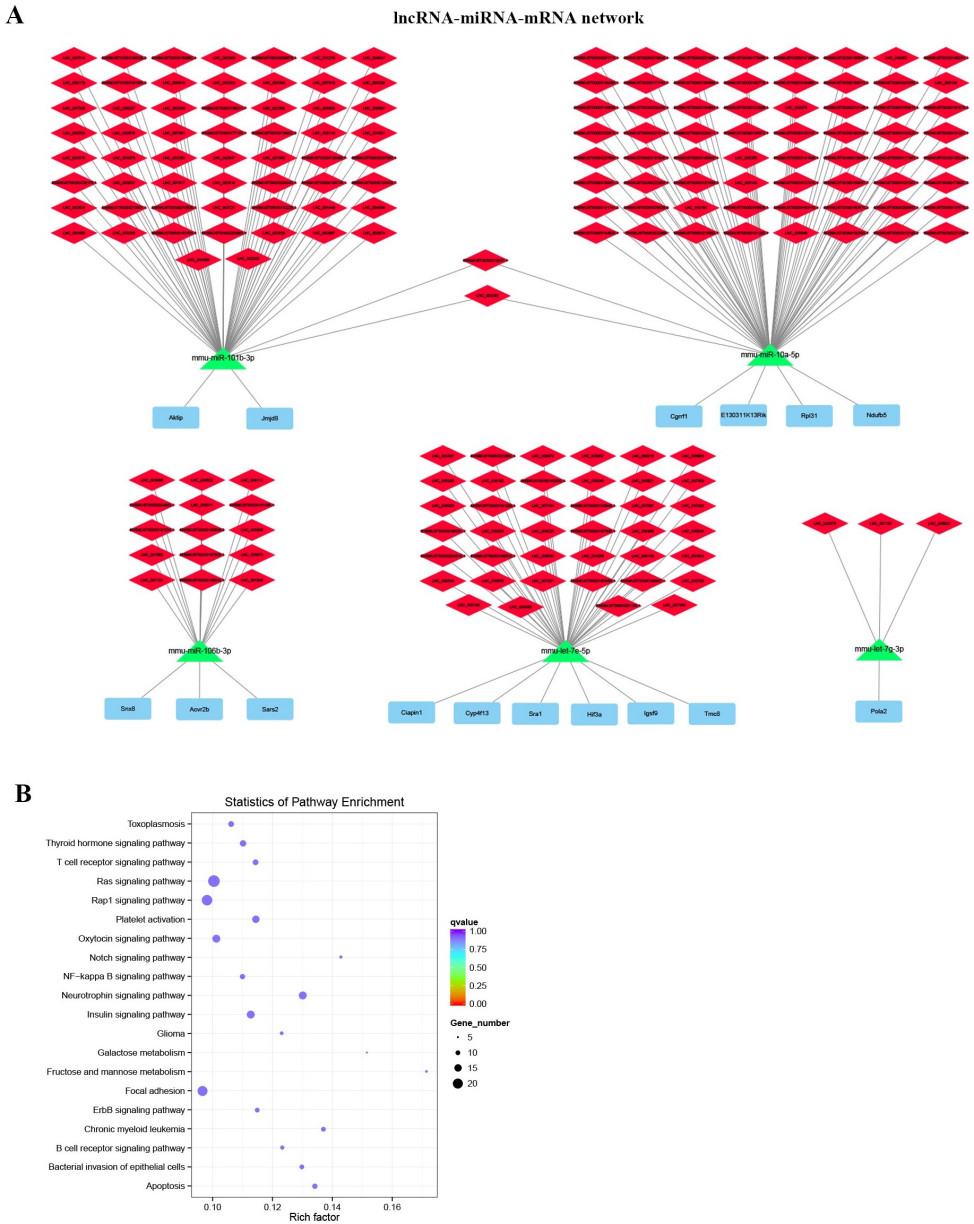
Construction of lncRNA-miRNA-mRNA network in ALI mouse model. (A) The lncRNA-miRNA-mRNA networks. (B) The bubble plots of KEGG pathway enrichment of DE mRNAs that involved in lncRNA-miRNA-mRNA network.

Based on the relationship of co-expression of lncRNA and miRNA, and co-expression of mRNA and miRNA, the lncRNA-miRNA-mRNA networks were constructed. The target lncRNAs of miRNAs were predicated by miRanda, and the correlation between lncRNA and miRNA were analyzed by Pearson’s correlation analysis (S16 Table). And the target mRNAs of miRNAs were predicated by miRanda, PITA, RNAhybrid database and the correlation between miRNAs and mRNAs were examined by Pearson’s correlation analysis (S17 Table). Then, 182 lncRNA-miRNA-mRNA networks were constructed (S18 Table). As shown in Fig 6A, 5 hub modules were identified based on the 5 miRNAs and 16 DE mRNAs with highest correlation. In addition, GO term analysis indicated that DE mRNAs in the lncRNA-miRNA-mRNA networks mainly associated with positive regulation of biological process (BP), intracellular part (CC) and macromolecular complex binding (MF) (S19 Table). KEGG pathway analysis demonstrated that DE mRNAs in the lncRNA-miRNA-mRNA networks mostly enriched in neurotrophin signaling pathway, apoptosis, chronic myeloid leukemia, insulin signaling pathway, platelet activation pathways according to the *P*<0.05 (Fig 6B, S20 Table).

### Construction of circRNA-miRNA-mRNA network in ALI mouse model

**Fig 7:**
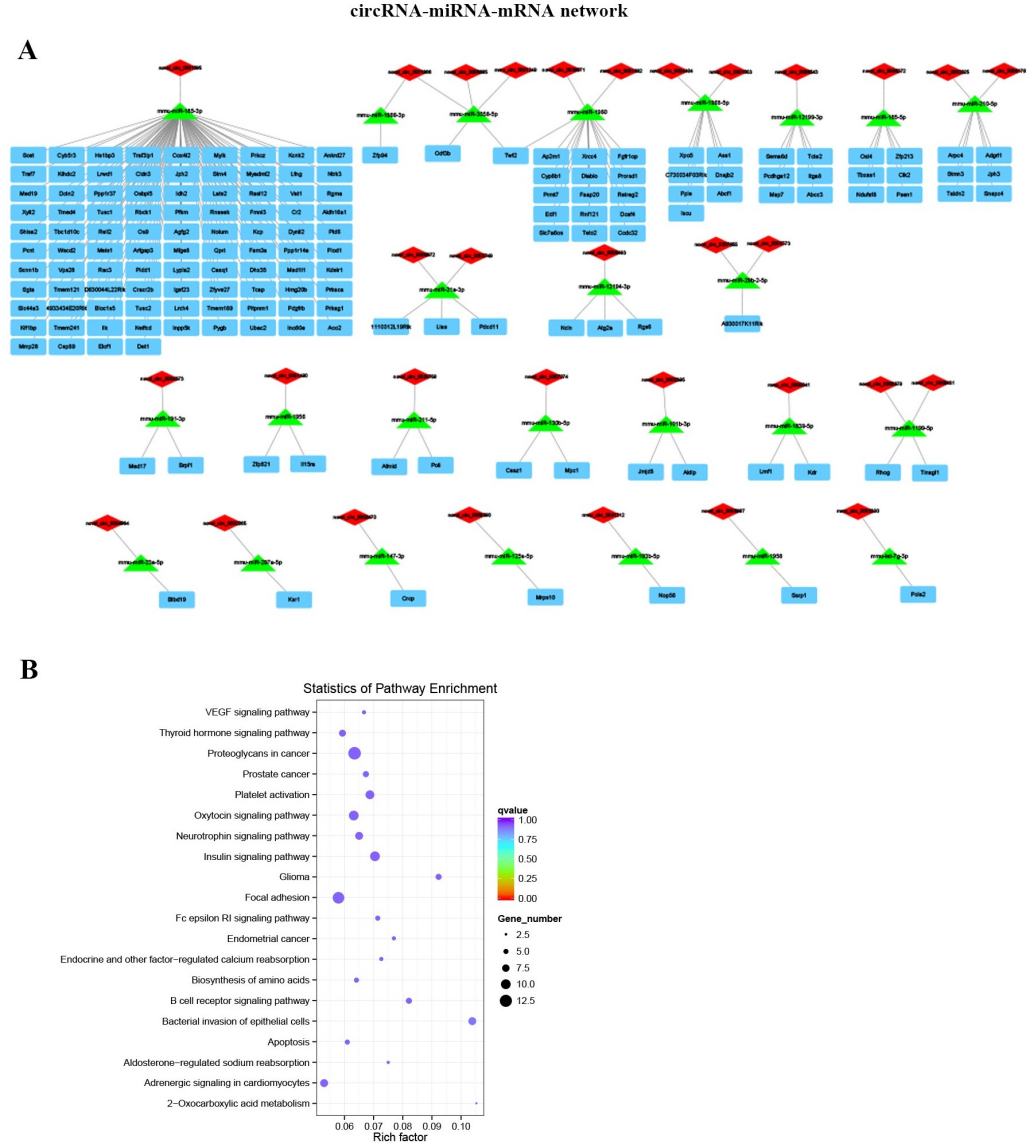
Established circRNA-miRNA-mRNA network in ALI mouse model. (A) Illustration of the circRNA-miRNA-mRNA networks. (B) The bubble plots of KEGG pathway enrichment of DE mRNAs that associated in circRNA-miRNA-mRNA network.

The correlation between miRNAs and circRNAs was confirmed by Pearson’s correlation analysis, the 32 circRNA-miRNA-mRNA networks were further established according to the 25 DE miRNAs and 32 DE circRNAs (Fig 7A, S21 Table). Furthermore, the GO analysis suggested that DE mRNAs in the circRNA-miRNA-mRNA networks were mainly involved in cellular protein modification process (BP), intracellular part (CC), and protein binding (MF) (S22 Table). And the KEGG pathway analysis indicated that DE mRNAs mainly involved in pathways including bacterial invasion of epithelial cells, proteoglycans in cancer, insulin signaling pathway and so on with P<0.05 (Fig 7B, S23 Table).

## Discussion

Genetic factors play the important roles in the pathogenesis of ALI, which accelerate the occurrence and development of ALI [23, 24]. Exploration more ALI-related genes have benefited for studying the potential molecular mechanism of ALI for early therapies. Therefore, in the present study, we constructed the LPS-induced ALI animal model and screened the aberrant expressed genes in LPS-induced ALI, we identified total 201 DE lncRNAs, 172 DE circRNAs, 62 DE miRNAs, and 3081 DE mRNAs I LPS-induced ALI. Then, 182 lncRNA-miRNA-mRNA network, and 32 circRNA-miRNA-mRNA networks were constructed. In addition, the biological function of target genes was identified based on GO annotation and KEGG pathway enrichment analysis. The Rap1 signaling pathway, galactose metabolism, HIF-1 signaling pathway and so on were involved in the development of ALI.

It known to us, the transcribed regions of the genome include ribosomal RNAs, transfer RNAs, messenger RNAs, ncRNAs [25]. Because of the alternative splicing, transcription start sites and termination sites are common events relate to the heterogeneity loci of transcriptional output both in coding- and non-coding RNAs [26]. Therefore, there exit large number of unknown genes affect the initiation and development of diseases. The rapidly development of NGS methods and technologies provided the reliable and efficiency approach to capture both coding and non-coding RNA and to quantify the differential expression of genes in cells, tissues, organ and even a whole body [27]. Not only screening the deciphering genome structure and function, but also identifying the genetic networks with potential cellular, physiological, biochemical, biological system and construction of the molecular biomarkers to response to diseases and pathogens [28]. Hence, the aberrantly expressed genes in LPS-induced ALI were screened and identified based on the whole transcription sequencing analysis.

LncRNAs have been demonstrated to involve in multiple biological and pathological processes in ALI. LncRNAs function as regulators in ALI through mainly regulating genes in transcriptional, post-transcriptional, and epigenetic levels with different mechanism. Such as, lncRNAs bind with target proteins in cis-acting elements or complexes form to regulate transcription [29]. And lncRNAs affect mRNA degradation, splicing and translation to regulate gene expression in post-transcriptional levels [30]. Moreover, lncRNAs modify the chromatin, imprint genome, and compensate dosage to modulate epigenetic levels [31]. Mostly, lncRNAs act as the ceRNAs sponge miRNAs to regulate the stable of target genes [32]. Therefore, to explore more lncRNA-miRNA-mRNA network in ALI is vital for understanding the pathological processes of ALI for example, lncRNA CASC9, MALAT1, NEAT1 regulate ALI development by sponging miRNAs [33–35].

Not only lncRNAs, but also circRNAs also play the vital role in ALI development. CircRNAs have a covalently stable closed loop structure and generated from exonic or intronic sequences [36]. circRNAs usually function as a sponger to sponge miRNAs to modulate splicing and transcription, and to modify their parental gene expression [37]. circRNAs act as the novel stars in regulation of the ALI, just a few circRNAs have been identified in ALI. There remain various circRNAs needed to be identified.

## Conclusion

In summary, our study provided 182 lncRNA-miRNA-mRNA networks and 32 circRNA-miRNA-mRNA networks involved in ALI. Our finding might provide a novel insight and a bulk of ALI-related genes for ALI research. However, there also some limitations in this study, first, there are lack of the validation experiments to validate the results in this study. Second, lack of the clinical samples to analyze and confirm the results. Therefore, we will validate the differently expressed ceRNA networks in ALI mouse model in the future study. Besides, we plan to harvest the ALI clinical samples to identify the differently expressed ceRNA networks.

## Supporting information

S1 Fig The pie charts of the distribution of lncRNAs in whole-transcriptome profiles of three pairs ALI and control samples. (TIF)

S2 Fig (A)-(C) Distribution and length of lncRNAs and mRNAs in whole-transcriptome profiles of ALI samples. (D) The cumulative distribution of conservative scores of lncRNAs. (E) The pie chart of subtypes of lncRNAs.
(TIF)

S3 Fig The Directed Acyclic Graph (DAG) plots for GO annotation about the target of lncRNAs. (TIF)

S4 Fig The Directed Acyclic Graph (DAG) plots for GO annotation about the target of mRNAs and target of miRNAs. (TIF)

S5 Fig The Directed Acyclic Graph (DAG) plots for GO annotation about the target of target of circRNAs. (TIF)

S6 Fig The top 200 lncRNA-miRNA-mRNA networks. (PDF)

S7 Fig The top 200 circRNA-miRNA-mRNA networks. (PDF)

S1 Table (XLS)

S2 Table (XLS)

S3 Table (XLS)

S4 Table (XLS)

S5 Table (XLS)

S6 Table (XLSX)

S7 Table (XLS)

S8 Table (XLS)

S9 Table (XLS)

S10 Table (XLS)

S11 Table (XLS)

S12 Table (XLS)

S13 Table (XLS)

S14 Table (XLS)

S15 Table (XLS)

S16 Table (XLSX)

S17 Table (XLS)

S18 Table (XLS)

S19 Table (XLS)

S20 Table (XLS)

S21 Table (XLS)

S22 Table (XLS)

S23 Table (XLS)
