# Supplementary figures and images for: Whole transcriptome analysis of the differential RNA profiles and associated competing endogenous RNA networks in LPS-induced acute lung injury (ALI)
Source: PLoS One. 2021 May 7;16(5):e0251359. doi: 10.1371/journal.pone.0251359 (PMC8104378; doi:10.1371/journal.pone.0251359)

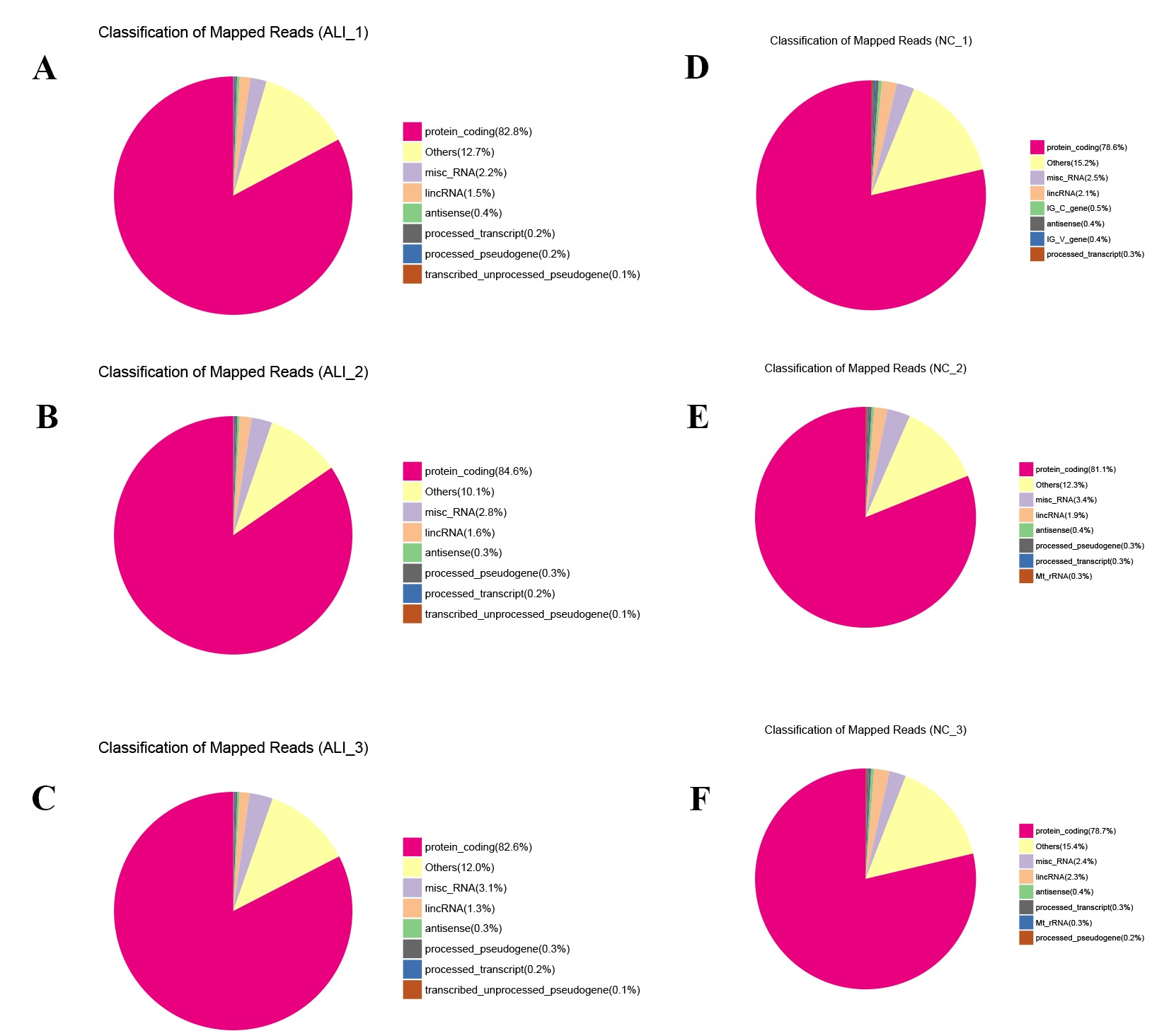

Supplement: S1 Fig — (TIF) [file pone.0251359.s001.tif]

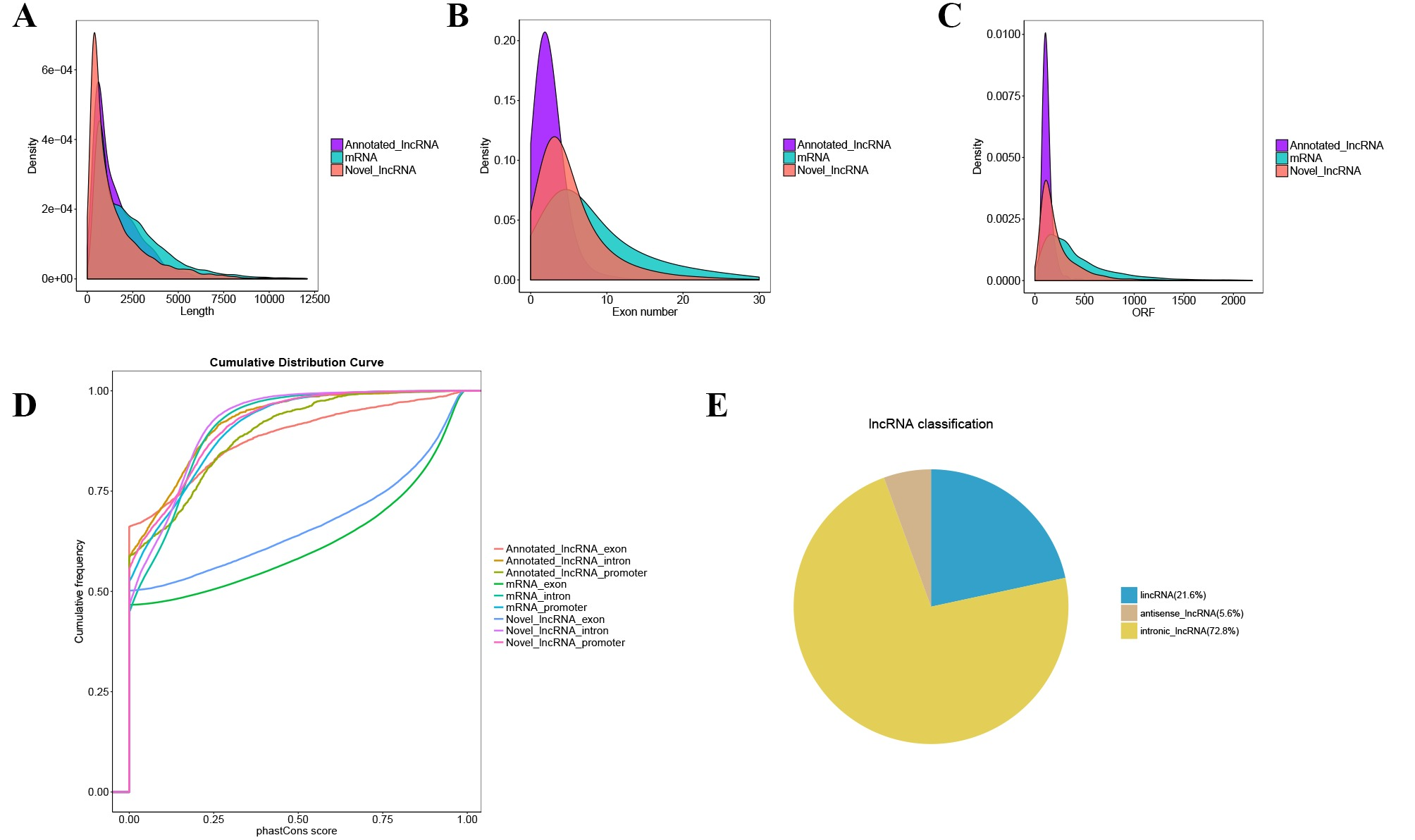

Supplement: S2 Fig — (A)-(C) Distribution and length of lncRNAs and mRNAs in whole-transcriptome profiles of ALI samples. (D) The cumulative distribution of conservative scores of lncRNAs. (E) The pie chart of subtypes of lncRNAs. (TIF) [file pone.0251359.s002.tif]

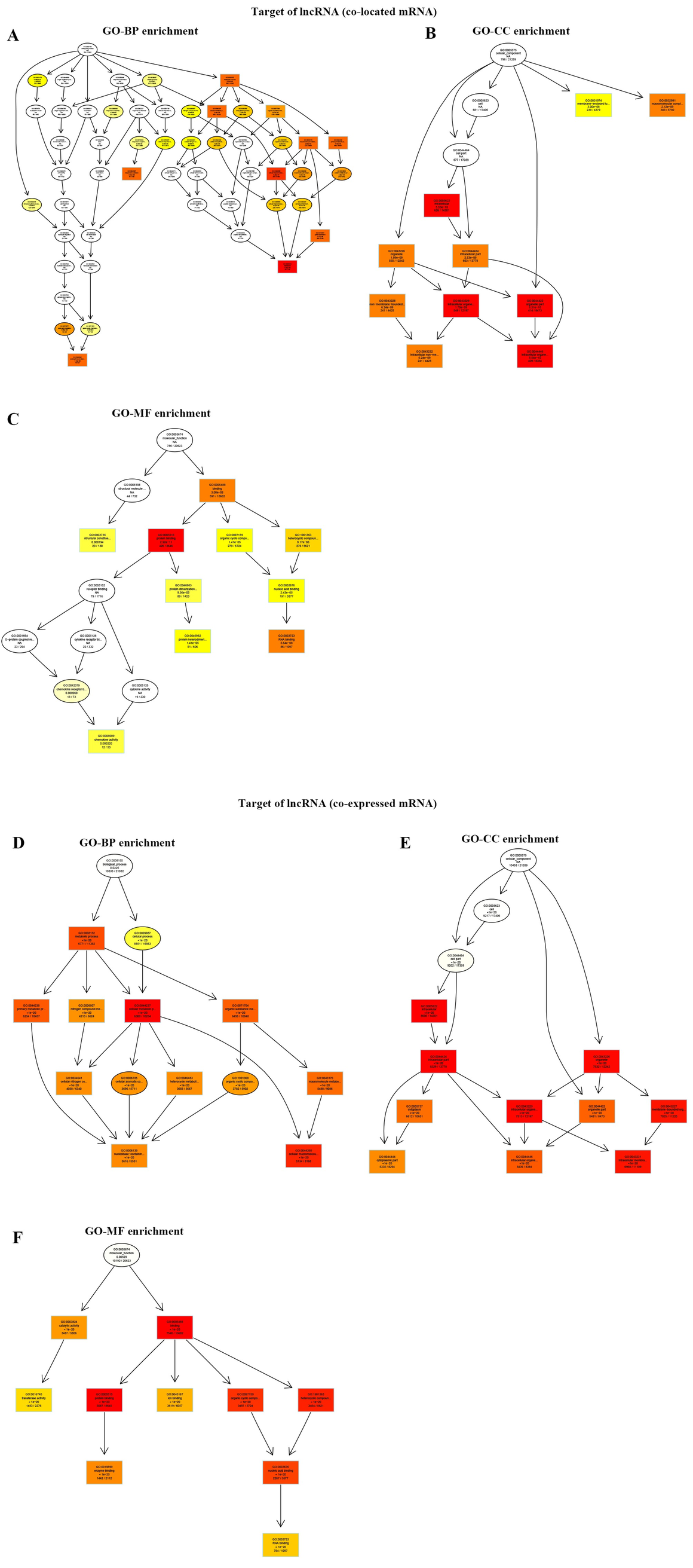

Supplement: S3 Fig — (TIF) [file pone.0251359.s003.tif]

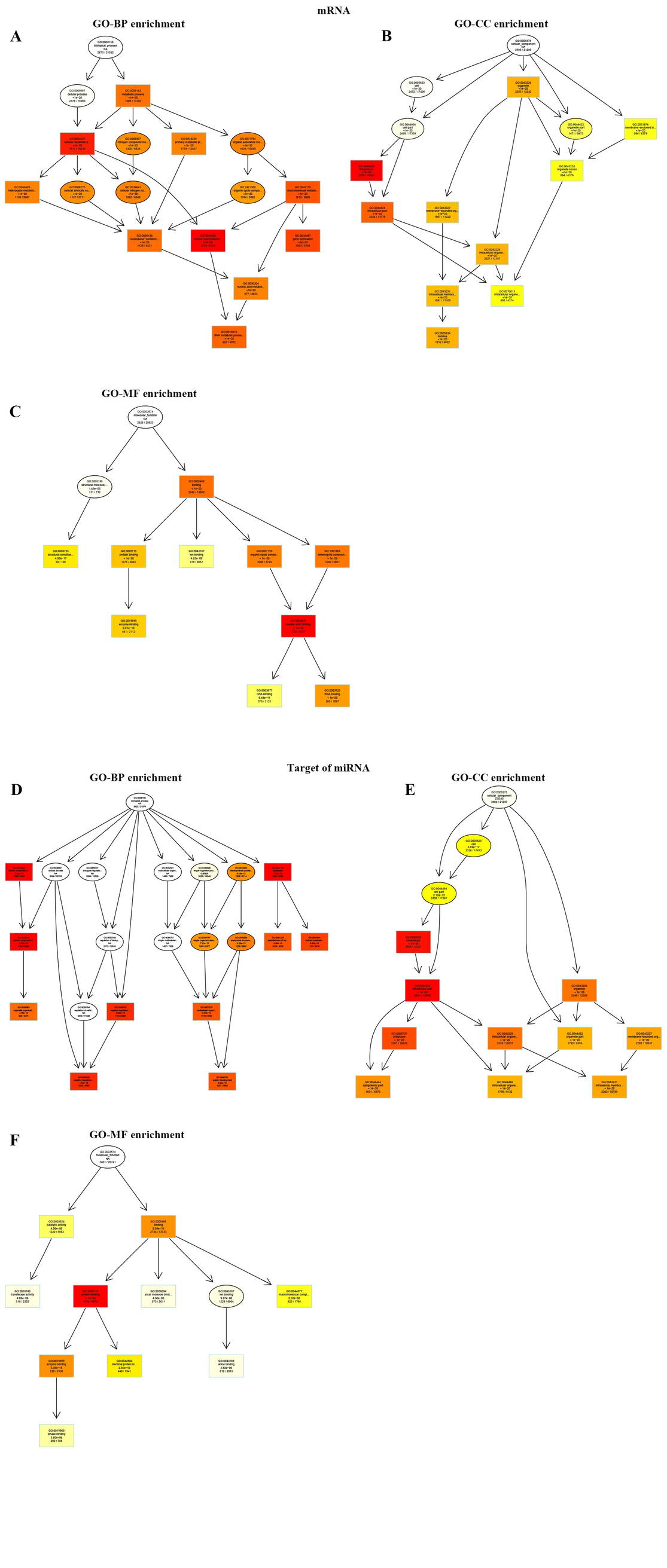

Supplement: S4 Fig — (TIF) [file pone.0251359.s004.tif]

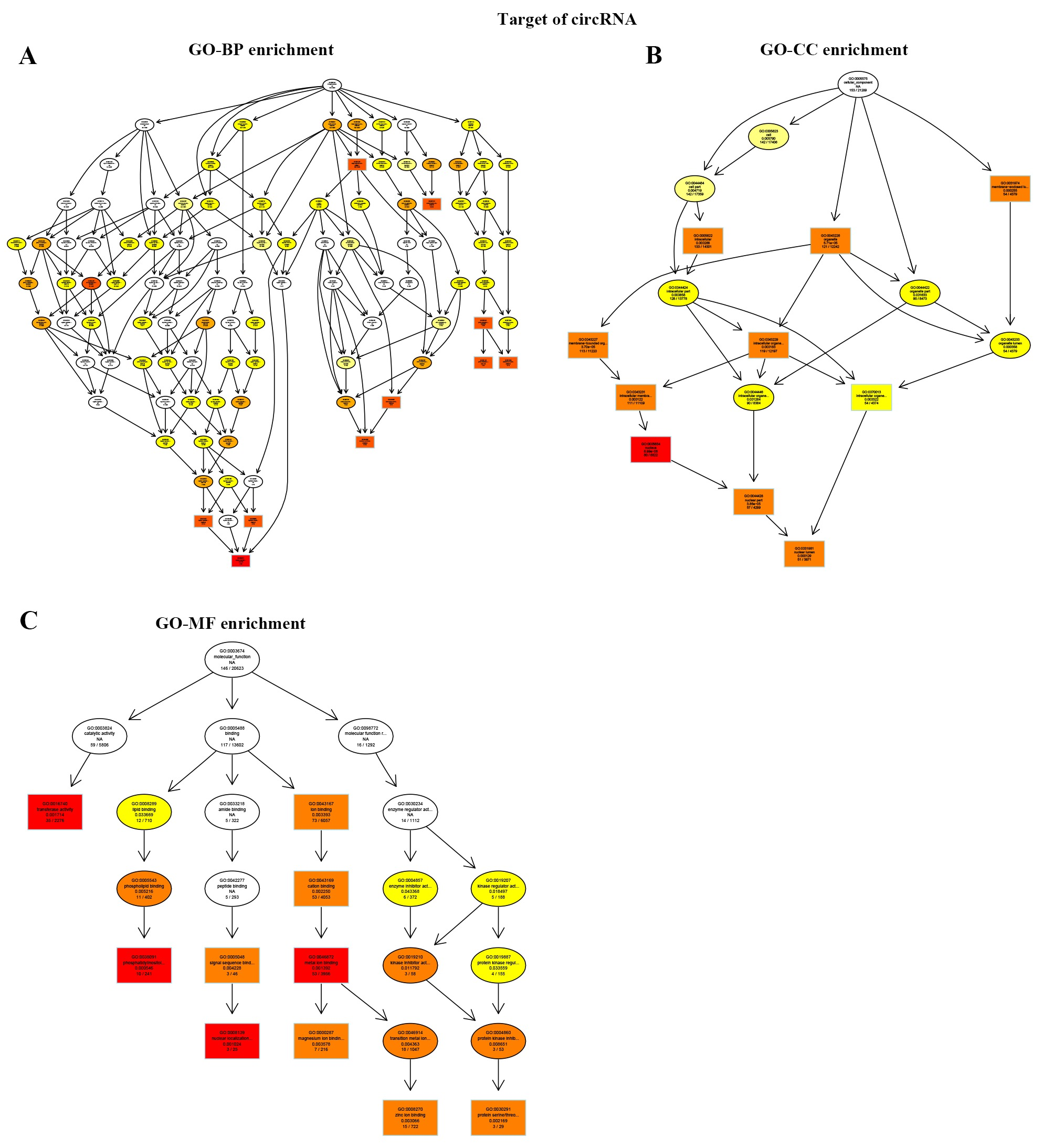

Supplement: S5 Fig — (TIF) [file pone.0251359.s005.tif]

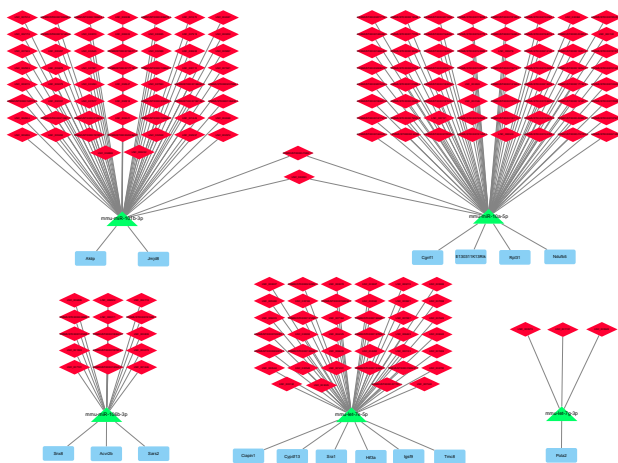

Supplement: S6 Fig — (PDF) [file pone.0251359.s006.pdf]

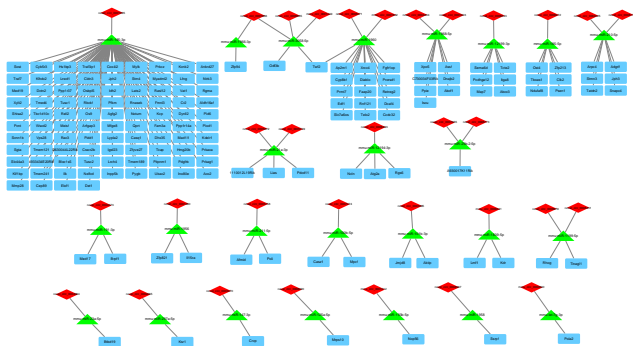

Supplement: S7 Fig — (PDF) [file pone.0251359.s007.pdf]
